# Comparison of SUV_max_ Values Obtained from F-18 FDG PET/CT and Cell-free DNA Levels Measured from Plasma in Oncology Patients

**DOI:** 10.4274/mirt.galenos.2019.60352

**Published:** 2019-06-24

**Authors:** Fatmanur Çelik, Yusuf Ziya Tan, Semra Özdemir, Fatma Sılan

**Affiliations:** 1 Çanakkale Onsekiz Mart University Faculty of Medicine, Department of Nuclear Medicine, Çanakkale, Turkey; 2 Çanakkale Onsekiz Mart University Faculty of Medicine, Department of Medical Genetic, Çanakkale, Turkey

**Keywords:** F-18 FDG PET/CT, SUVmax, cell-free DNA

## Abstract

**Objectives:**

The aim of this study was to compare the quantitative value of standardized uptake value (SUV) SUV_max_ obtained from F-18 FDG positron emission tomography/computed tomography (PET/CT) imaging of oncology patients with the cell-free DNA (cfDNA) amounts measured in plasma of patients and thus investigate if cfDNA is a significant marker to identify the presence of malignancy in the early period.

**Methods:**

A total of 184 patients were included in the study. The clinical, histopathologic, laboratory and treatment parameters were extracted from patient files. SUV_max_ and cfDNA quantities were assessed.

**Results:**

There was no statistically significant difference in plasma cfDNA values between patient and control groups. The comparison of SUV_max_ and cfDNA values in the study showed that there was a weak correlation between SUV_max_ and cfDNA. There was a significant difference between tumor size and SUV_max_ values. However, there was no statistically significant difference between tumor size and cfDNA.

**Conclusion:**

cfDNA measurements in the blood as a screening test have provided hope for early diagnosis and monitoring of cancer patients. Comparison of cfDNA levels obtained from plasma and quantitative parameters from PET/CT images of oncology patients in detailed advanced studies with larger patient series are required.

## Abstract (tr)

**Amaç:**

Bu çalışmanın amacı, onkoloji hastalarında F-18 FDG pozitron emisyon tomografi/bilgisayarlı tomografi (PET/BT) görüntülemelerinden elde edilen standart tutulum değeri (SUV) SUV_maks_’ın, hastaların plazmalarında ölçülen serbest DNA (cfDNA) miktarları ile kantitatif değerini karşılaştırmak ve böylece cfDNA’nın erken dönemde malignitenin varlığını tanımlamak için önemli bir belirteç olup olmadığını araştırmaktır.

**Yöntem:**

Çalışmaya toplam 184 hasta dahil edildi. Klinik durum, histopatolojik, laboratuvar ve tedavi parametreleri hasta dosyalarından araştırıldı. SUV_maks_ değeri ve cfDNA miktarları karşılaştırıldı.

**Bulgular:**

Hasta ve kontrol grubu arasında plazma cfDNA değerleri arasında istatistiksel olarak anlamlı fark yoktu. Araştırmada SUV_maks_ ve cfDNA değerlerinin karşılaştırılmasının sonucunda SUV_maks_ ve cfDNA arasında zayıf bir korelasyon vardı. Tümör boyutu ve SUV_maks_ değerleri arasında anlamlı bir fark bulundu. Bununla birlikte, tümör boyutu ve cfDNA arasında belirgin bir istatistiksel farklılık saptanmadı.

**Sonuç:**

Basit bir tarama testi olarak kullanılabilecek olan cfDNA ölçümleri kanser hastalarının erken tanı ve takiplerinde umut vaad etmektedir. Bu nedenle onkoloji hastalarının plazmalarından elde edilen cfDNA düzeyi ve PET/BT görüntülerinden elde edilen kantitatif parametrelerin karşılaştırıldığı daha büyük hasta serilerinde ve detaylı ileri çalışmalara ihtiyaç vardır.

## Introduction

Cancer is an important health problem. It is the second most common cause of death in the world generally, after cardiovascular diseases. It is predicted that in future years the incidence will significantly increase (1).

Survival after cancer is linked to factors such as tumor stage at time of diagnosis, form of treatment, general state of the patient, and morphologic and molecular characteristics of the tumor. As a result, early detection of cancer has great importance in preventing mortality and morbidity linked to cancer. Though there are advances in the diagnosis and treatment of some cancer types, early diagnosis and treatment of cancer continues to be a significant problem.

In recent years, the importance of the nuclear medicine imaging method positron emission tomography/computed tomography (PET/CT) used for diagnosis, staging and monitoring the treatment of various cancers has increased worldwide (2).

PET/CT images are assessed qualitatively and semi-quantitatively. The most commonly used parameter in semi-quantitative evaluation is the standardized uptake value of F-18 FDG called “standardized uptake value” (SUV). The SUV value represents the amount of radioactivity accumulated per gram of tissue (3).

In addition to conventional imaging modalities, biological markers are being used to distinguish tumor cells from normal cells for early diagnosis (4).

During the last years, circulating cell-free DNA (cfDNA) in the blood of healthy and cancer patients gained increasing attention. It was understood in the late 1980s that the DNA in the circulation has neoplastic property and reflects the biological character of the tumor (5,6).

The study relies on physical and biological properties of DNA that differ in normal tissues as compared to tumors.

However, although tumor markers found in serum in patients and specific to some types of cancer are routinely used for early identification of oncologic diseases, due to limited specificity and sensitivity the desired results have not been reached for early diagnosis. Consequently, its routine application is still not recommended.

The aim of this study was to compare the quantitative value of SUV_max_ obtained from full body PET/CT imaging of oncology patients with cfDNA amounts measured in plasma of patients, and thus investigate whether cfDNA is a significant marker to identify the presence of malignancy in the early period.

The study was approved by Çanakkale University Ethics Committee (protocol number: 204-14). Informed consent was obtained from all participants.

## Materials and Methods

### Patient Selection

The study was prospective and was begun after receiving ethics committee approval. It included 184 oncology patients (87 females, 97 males) directed for F-18 FDG PET/CT imaging from January 2015-February 2016 and a control group of 92 people comprising 57 females and 35 males. Ninety-two people was enrolled as a control group. Patients who had no known oncological disease but were suspected to have laboratory and clinically were included in the study. Study patients did not have any comorbid diseases.

The clinical, histopathologic, laboratory and treatment parameters were extracted from patient files. The patients’ age, gender, weight, height, smoking habit, accompanying diseases, date of diagnosis, diagnosis methods, histologic types, stages, tumor diameter, number and location of metastases, and chemotherapy and radiotherapy histories were investigated.

### PET/CT Procedure

Imaging of patients was completed with a Biograph Duo LSO F-18 FDG PET/CT scanner (Siemens, Germany). All patients received routine PET/CT imaging protocol. According to this protocol, patients were requested to avoid excessive physical exercise and exposure to cold two days prior to imaging, and starve for at least six hours. Before imaging, all patients had glucose measurements from capillary blood and F-18 FDG PET/CT imaging was delayed in those with serum glucose levels above 180 mg/dL to allow blood sugar regulation. Patients with appropriate blood sugar levels were injected with 8-12 mCi F-18 FDG intravenously with the aid of an angiocath. After the injection, patients rested in a calm and comfortable environment without speaking or moving for 45-60 minutes to provide biodistribution of the radiopharmaceutical and ensure ideal tumor uptake. At the end of the waiting period, patients emptied their bladders and laid on the PET/CT scanner bed in the supine position with arms at the sides. Initial guideline topogram images were obtained, non-contrast CT images were taken for the body regions from the vertex to 1/3 proximal thigh followed by PET images. The patient’s PET/CT images were taken with mean 7-8 bed positions and 2 mm slices and were completed in about 25 minutes.

The PET/CT images of all patients were reported within the framework of routine evaluation procedure by at least one nuclear medicine specialist and a senior nuclear medicine assistant. Within this procedure, multiplanar PET, CT and PET/CT fusion slices with and without attenuation correction and maximum intensity projection PET images were investigated on an LCD monitor using a computer software program (esoft Workstation, Syngo MI, Siemens). Evaluation was made considering the clinical history obtained from patient files and direct interviews with the patient, current complaints, conventional imaging findings, biopsy results and previous operation history. Lesions identified on PET/CT were primarily visually assessed. For quantitative assessment, SUV_max_ values were used. SUV_max_ values were measured according to region of interest and automatically calculated by the computer. The SUV_max_ value was measured in the lesion with highest F-18 FDG uptake among all positive lesions.

### cfDNA Measurement

Each case had 10 mL venous blood sample obtained from the forearm and taken in ethylene diamine tetra acetic acid tubes which were sent to the laboratory. Without delay, blood samples were centrifuged at 3800 rpm for 10 minutes. The supernatant was transferred to a new tube and centrifuged at 3000 rpm for 10 minutes. Later, plasma samples of 1 mL each were distributed to cryo tubes and stored at -20 C until use.

Automatic DNA isolation was completed with a MagNA pure nucleic acid isolation kit in accordance with total nucleic acid plasma protocol. Using 400 µL plasma samples the elution buffer amount was determined as 50 µL. The obtained samples were spectrophotometrically measured at 260 nm and 280 nm wavelengths and then DNA amount and DNA purity levels were measured as ng/mL with nanodrop.

### Statistical Analysis

Analysis of the study data used SPSS for Windows 22.0 packet program. The Shapiro-Wilk test was used to test whether data had normal distribution or not. Data without normal distribution had the Kruskal-Wallis test used to compare more than two independent groups. If significant differences were found, the groups were compared in pairs with the Mann-Whitney U test. The Mann-Whitney U test was used for comparison of two independent groups without normal distribution. Variables without normal distribution are given as median (minimum-maximum) values. The significance level for statistical analysis was taken as p<0.05.

## Results

### Patient Characteristics

The patients in the study group were 87 females (47.3%) and 97 males (52.7%) with an age range for the total of 184 patients of 25 to 89 years and mean age of 53.38±17.98 years. The control group comprised 57 females (62.0%) and 35 males (38.0%) for a total of 92 patients with ages ranging from 19 to 86 years and mean age calculated as 36.5±12.98 years (Table 1).

When the study group and control group patients were divided into two groups as those below the age of 50 and above; there were 25 patients in the study group (13.6%) below the age of 50 with 159 patients (86.4%) above 50 years of age. In the control group, there were 64 cases (69.6%) below the age of 50 and 28 cases (30.4%) above the age of 50. In terms of age distribution above and below the age of 50 in the two groups, there was a statistically significant difference between patients in the control and study groups (p<0.001, Table 2).

When the patients were compared in terms of smoking habit, 99 cases in the patient group (53.8%) smoked while 40 cases in the control group (43.5%) smoked. There was no statistically significant difference between the two groups in terms of smoking history (p=0.136, Table 2).

### cfDNA Measurements in Two Groups

Evaluation of the plasma cfDNA values identified the mean cfDNA value in the patient group as 8.8 ng/mL (0-50) and in the control group as 8 ng/mL (0.5-21). Comparison between the groups did not reveal a statistically significant difference (p=0.405, Table 3).

### Comparison of Oncologic Subtypes According to cfDNA

When 184 patients are compared in terms of oncologic subtypes, operation history and treatment, there was no statistically significant difference between the two groups. When patients participating in the study had oncologic subtypes, operation and treatment histories compared with plasma cfDNA levels, there was no statistically significant difference (Table 4). Additionally, there was no statistically significant difference in terms of operation history, chemotherapy and radiotherapy treatment. However; lung, cervix, thyroid and pancreas cancers were identified to have higher cfDNA values as compared to other types (Table 4).

### Comparison of Tumor and Metastatic Lesion SUV_max_ Values and cfDNA Measurements

**Figure 1:**
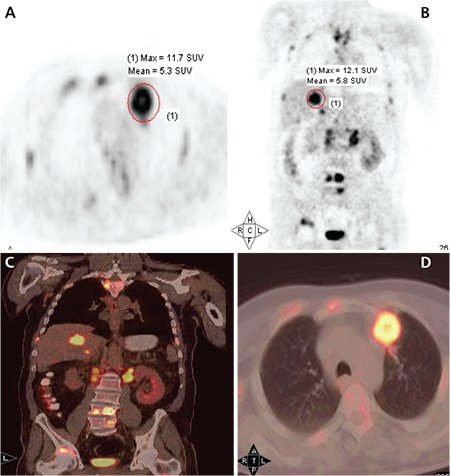
A patient with non-small cell lung carcinoma. The tumor in the left upper lobe shows F-18 FDG uptake (SUV_max_: 11.7) as well as liver metastasis (SUV_max_: 12.1). Coronal maximum intensity projection (A) and axial PET images (B) and axial PET/CT images (C), Coronal fused PET/CT images (D). The amount of cfDNA obtained from the patient’s plasma was measured as 5 ng/mL. Primary tumor and liver metastasis lesions’ SUV_max_ values and cfDNA values were compared. There was no significant difference between primary tumor and metastasis SUV_max_ and cfDNA values

SUV_max_ measured in tumor and metastatic lesions were compared along with plasma cfDNA values (Figure 1). There was no statistically significant difference between the two groups in terms of tumor and metastasis presence (p=0.497, Table 5). However, comparison between the groups in terms of presence of malignant lesions and metastasis with SUV_max_ values identified a statistically significant difference in patients with metastasis as compared to patients without metastasis (p=0.049, Table 5).

### Comparison of SUV_max_ Value and cfDNA Measurements According to Tumor Size

The SUV_max_ values and cfDNA values of patients according to tumor size are presented in Table 6. Accordingly, the mean SUV_max_ values of lesions with tumor size <2 cm were identified as 2.4 (0-34.4), as 10.4 (3.20-53) for masses 2-6 cm and as 13.35 (3.3-34.2) for masses ≥6 cm. Statistically, there was a significant difference identified between tumor size and SUV_max_ values. There was no clear statistical difference identified between tumor size and cfDNA.

## Discussion

Several studies in the literature reported an increase of cfDNA in various types of cancer (7). A significant portion of these studies stated there were increased amounts of cfDNA in oncology patients as compared to normal patients healthy human beings (8). However, there are only a few comparative studies on cfDNA and tumor metabolic activity. We identified a weak correlation between SUV_max_ values and cfDNA. Furthermore, our results showed higher cfDNA values in lung, cervix, thyroid and pancreas cancers as compared to other type of malignancies.

A study on the correlation between ovarian cancer and cfDNA levels reported increase in cfDNA levels prior to epithelial ovarian cancer operations (9).

The 2005 study on cases with thoracic malignancy by Herrera et al. (10) is noteworthy as they did not observe a significant difference in the plasma cfDNA levels of healthy individuals, gastroesophageal reflux patients, esophageal and lung cancer patients. Similarly, in our study, there was no significant difference in cfDNA levels between the control and the patient groups. However, the results of this study identified that cfDNA levels were high in metastatic lung and cervix cancer.

Some studies on the correlations between cancer and risk factors have stated that cancer risk increases with age.

In a 2017 study on cfDNA amounts and mutations in cancer patients and healthy controls, Chen et al. (11) stratified the healthy controls according to gender and age (<50 - ≥50) and did not find a significant difference in cfDNA levels between groups. Similarly, in our study, age (<50 - ≥50) and gender were not observed to cause a difference in cfDNA amounts.

Kim et al. (12) reported that cfDNA amounts were higher in the non-smoking patient group as compared to smokers in a cohort of gastric carcinoma patients. In our study, comparison of smoking and non-smoking patients and control groups did not reveal a significant correlation between cfDNA levels with smoking in both groups.

Some literature studies have stated that cfDNA concentrations in healthy subjects ranged between 0 and 100 ng/mL, whereas in cancer patients the concentration in plasma or in serum ranged between 0 and 1000 ng/mL (13).

It is not known whether this broad range of cfDNA levels is linked to normal physiologic variability or to chronic or sub-clinical pathologic situations. It is likely that body mass index, presence of a sub-clinical disease at the time of measurements, and chronic disease may affect cfDNA levels. In our study, cfDNA levels were measured according to the current disease of the patient. Previous diseases and blood markers of these diseases were not measured. However, there was no significant difference between the oncologic patient and control group in terms of cfDNA levels.

A broad-scale study of oncology patients and healthy cases found a significant difference in cfDNA concentrations between the groups included in the study; however, no cut-off value could be determined for the use of cfDNA in cancer diagnosis screening (14). In our study, we did not determine a cut-off value between the patient and control groups. The measurements could not be standardized due to reasons such as sampling at different times, and including various operators although the same technique was used. There was also no access to broad patient-linked investigations, thus an appropriate reference interval could not b determined.

In a study by Heitzer et al. (15), cfDNA belonging to the tumor was not found in the plasma at measurable intervals in some of the metastatic cancer patients.

In our study, there was no statistically significant difference identified in terms of cfDNA between metastatic and nonmetastatic disease. Comparison of the groups in terms of SUV_max _values in the presence of malignant lesions and metastatic disease identified a statistically significant difference between patients with metastasis and those without. As only a small portion of the total cfDNA in the circulation belongs to the tumor, identification of cfDNA at undetectably low levels in metastatic carcinoma patients appears possible, although it contradicts the literature. There are many studies indicating that plasma DNA levels correlate with tumor size, degree of tumor invasion, disease stage, survival and progression of disease with treatment.

Nygaard et al. (16), in a 2014 study evaluating the correlation between tumor load and cfDNA by using PET/CT in non-small cell lung cancers, did not find a correlation between metabolic tumor volume and tumor lesion glycolysis with cfDNA. Similarly, in our study, while there was an increase in SUV_max_ values linked to increased tumor volume, this increase did not appear to correlate with cfDNA.

### Study Limitations

There are several problems in evaluating cfDNA such as standardization of assays, isolation technologies, standards, assay conditions, and specificity and sensitivity rates (17). Blood collection, transport time and storage conditions should be optimized during the study.

Firstly, the technique used to measure cfDNA and thus the determined cfDNA concentrations may be different. As a result, it is very difficult to define a cut-off value to distinguish benign and malignant diseases. Moreover, it is not known whether this broad interval of cfDNA levels is normal physiologic variability or linked to chronic or subclinical pathologic situations. It appears probable that body mass index, current subclinical diseases and chronic diseases may affect cfDNA levels during measurement. In our study, patients had cfDNA levels measured based on current diseases. Previous diseases and markers of these diseases in the blood were not measured. Nevertheless, there was no significant difference observed in cfDNA levels between the oncologic patient and control group.

Secondly, there are some difficulties related to how to determine the sensitivity and specificity of molecular tumor markers at clinical levels. It is difficult to compare different research groups due to the use of different nucleic acid isolation techniques for plasma and serum. Though this study used the same technique, we identified different values linked to different personnel. As a result, we believe it is necessary to obtain nucleic acid automatically with certain standards to provide common use of serum/plasma DNA in the future.

## Conclusion

Currently, there is no diagnostic method that can be used for early diagnosis of cancer and evaluating response to treatment alone. Conventional imaging methods are extremely important in the diagnosis and treatment of oncology patients. However, a range of problems may be experienced due to ionizing radiation and assessment errors. As a result, there is a need for easy-to-use, simple additional screening methods. With this aim, cfDNA measurements in the blood for use as a simple screening test have provided hope for early diagnosis and monitoring of cancer patients in recent years. As a result, there is a need for comparison of cfDNA levels obtained from plasma and quantitative parameters from PET/CT images of oncology patients in more detailed advanced studies with larger patient series.

## Figures and Tables

**Table 1:**
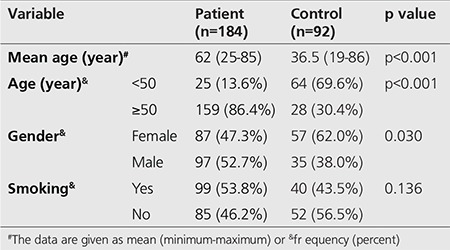
Patients characteristics-mean age and number of patients for each group

**Table 2:**
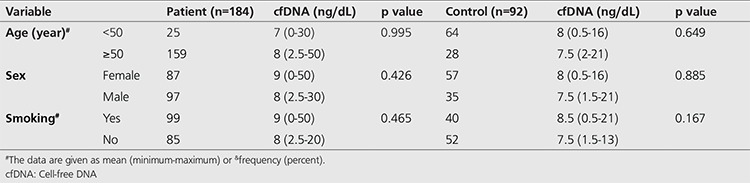
Comparison of the patient and control group’s demographic data with cell-free DNA quantities

**Table 3:**
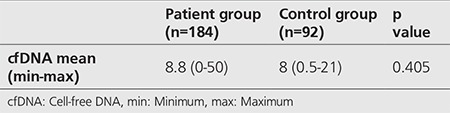
Comparison of cell-free DNA values of groups

**Table 4:**
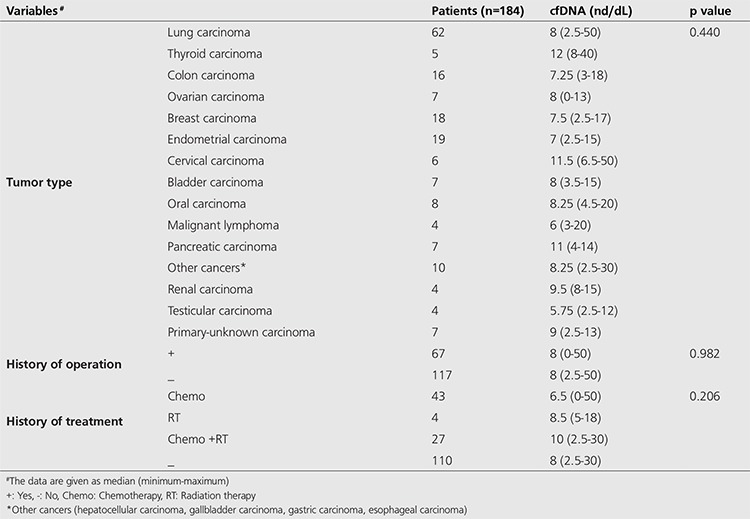
Comparison of cell-free DNA amount according to tumor type

**Table 5:**
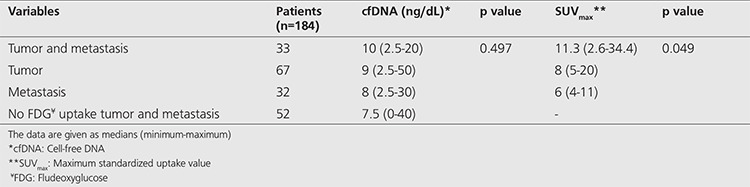
Comparison of tumor and metastasis presence with cell-free DNA and SUV_max_ values in oncologic patients

**Table 6:**
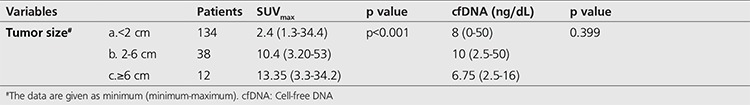
Comparison of tumor size, SUV_max_ and cell-free DNA values in oncologic patients
